# Carpal tunnel syndrome caused by tophi deposited under the epineurium of the median nerve: A case report

**DOI:** 10.3389/fsurg.2022.942062

**Published:** 2023-01-06

**Authors:** Wenzhong Zhang, Qingbo Feng, Jiaxiang Gu, Hongjun Liu

**Affiliations:** ^1^ Department of Hand and Foot Surgery, Northern Jiangsu People's Hospital, Clinic Medical College of Yangzhou University, Yangzhou, China; ^2^ Department of Liver Surgery and Liver Transplantation Centre, West China Hospital, Sichuan University, Chengdu, China

**Keywords:** carpal tunnel syndrome, gout, hyperuricemia, surgery, tophi, case report

## Abstract

**Introduction:**

Usually caused by compression of the wrist's median nerve, carpal tunnel syndrome (CTS) is one of the most common types of peripheral neuropathy. Tophi deposited under the epineurium of the median nerve compress the median nerve, leading to CTS, which is very rare.

**Case presentation:**

We report a 64-year-old man with a history of tophaceous gout who presented with typical CTS symptoms and was admitted to our hospital. A physical examination revealed swelling over the right volar aspect of the carpal region, and he was unable to flex due to subcutaneous rigidity. Tinel's sign and Phalen's maneuver were positive. Electrophysiological studies confirmed the diagnosis of CTS. A carpal tunnel release and surgery to remove the gouty tophus of the right wrist were performed when serum uric acid levels were within normal limits (5.8 mg/dl). During the operation, tophi deposited under the epineurium of the median nerve were found, and the tophi were completely removed. Operative findings confirmed the diagnosis of CTS due to gout. The patient recovered uneventfully without signs of recurrence of gout and CTS symptoms during a 1-year follow-up period.

**Conclusion:**

A gouty tophus is an uncommon cause of CTS, and CTS may be caused by gouty tophi if there is evidence of extrinsic compression of the median nerve or symptoms emanating from the carpal tunnel.

## Introduction

Carpal tunnel syndrome (CTS) is caused by compression of the median nerve as the most common upper limb peripheral nerve entrapment syndrome (1). Even though almost 50% of cases of CTS are idiopathic, recognized causes of CTS include tissue infiltration, tissue edema, tissue inflammation, hemorrhage after injury, and congenital variations, such as an abnormal muscle, tendon, or persistent median artery (2–8). In addition to the radiocarpal joint, the flexor tendons, tendon sheaths, and the carpal tunnel floor, gouty tophi deposits can accumulate in various structures, resulting in nontraumatic compression of the median nerve (2).

We report a very rare case of CTS caused by tophi deposited under the epineurium of the median nerve and analyze its diagnosis and treatment. Gouty tophi are an uncommon cause of CTS, and the incidence is 0.6% approximately (9). Also, most of the cases are caused by gouty tophi in flexor tendons (10–12). This report presents a rare case of CTS caused by gouty tophi deposited under the epineurium of the median nerve and successfully treated by surgery. To our knowledge, there is the first report with a picture of tophaceous gout deposited under the epineurium of the median nerve.

## Case presentation

**Figure 1:**
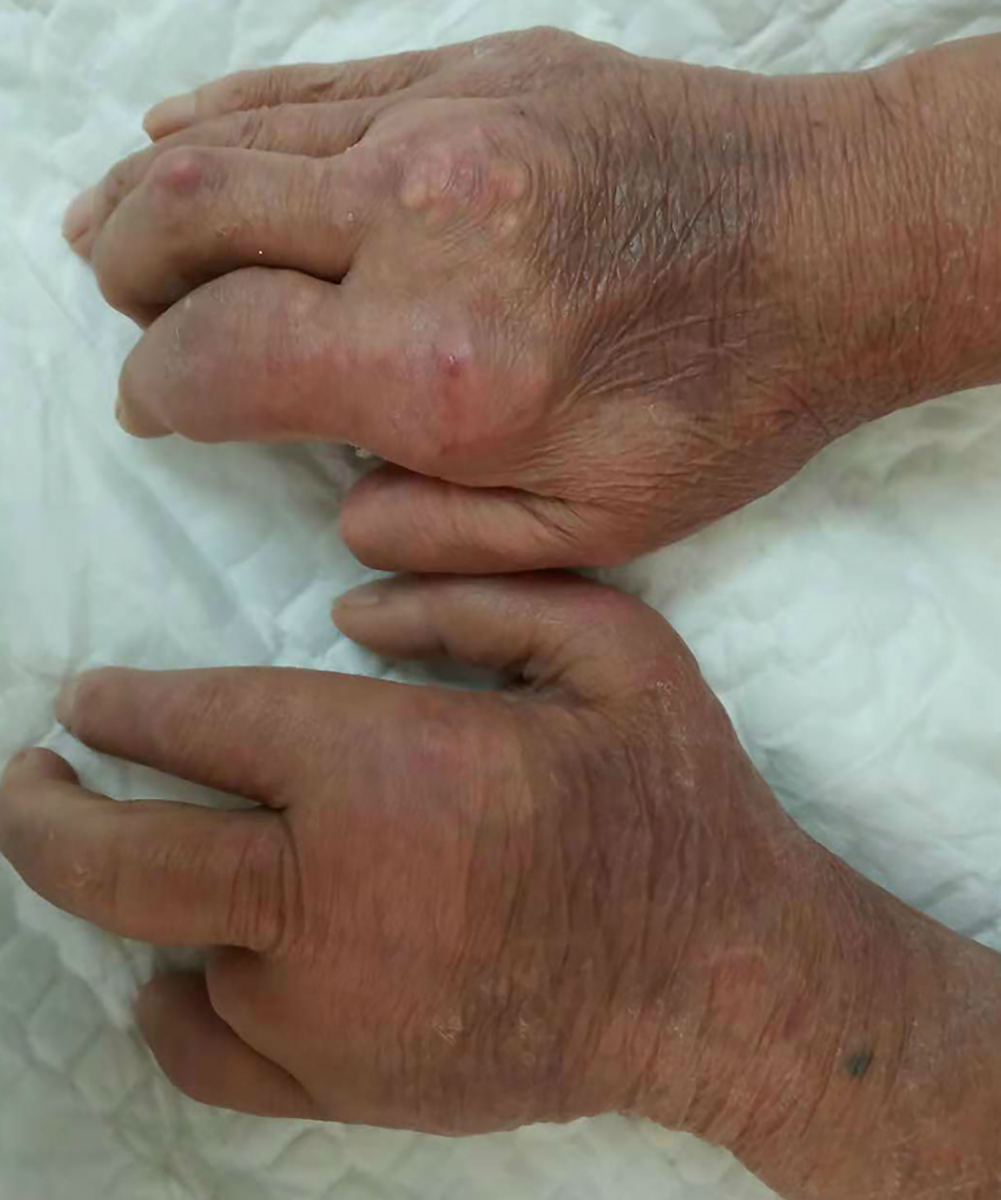
Preoperative examination showing that gout nodules of different sizes could be touched in both hands.

**Figure 2:**
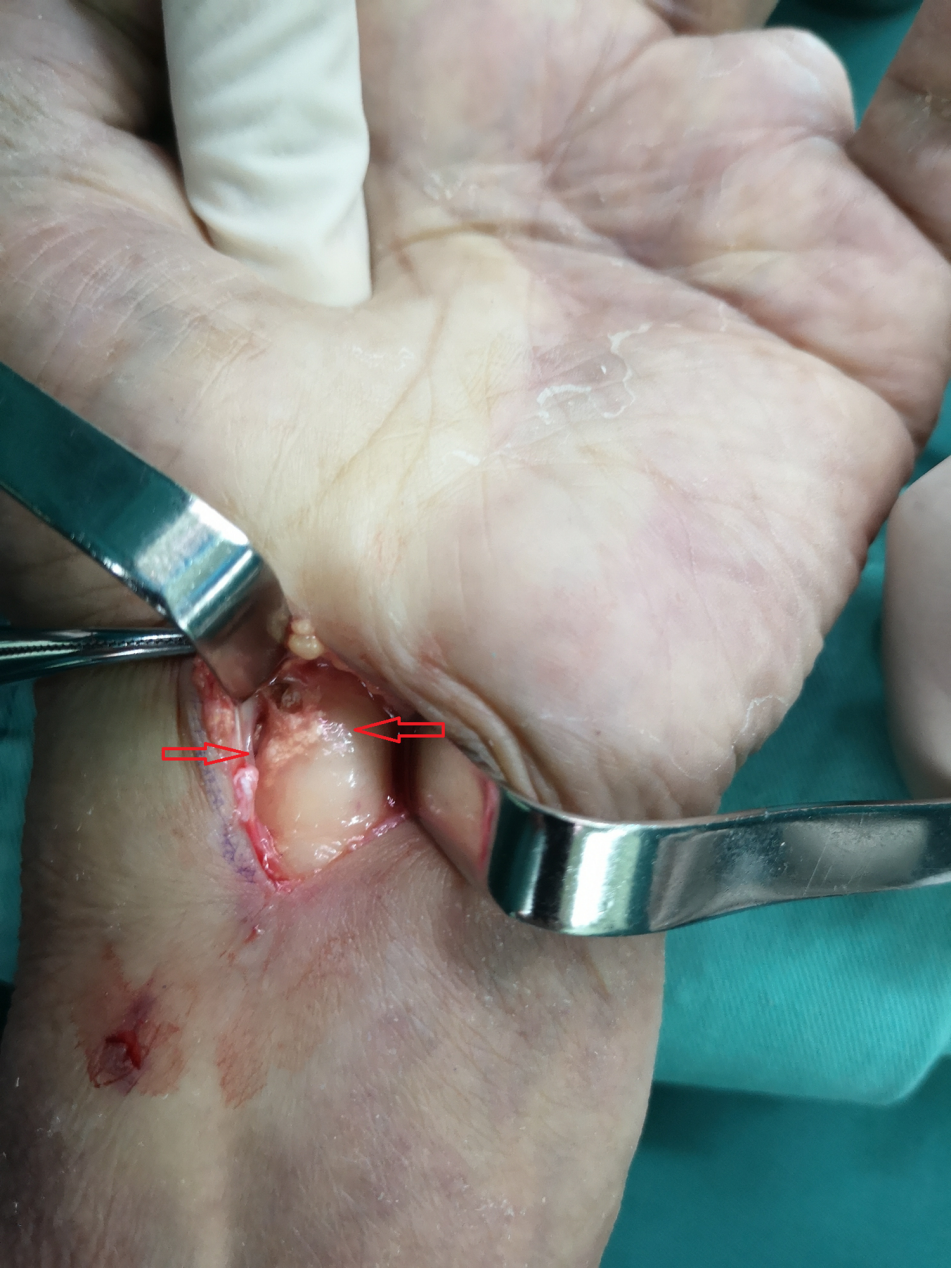
Operative findings: gout tophi (red arrows) deposited under the epineurium of the median nerve.

The reporting of this study conforms to CARE guidelines. A 64-year-old men had a history of gout for 27 years without treatment and presented with numbness and tingling in his thumb, index, and middle fingers. The symptoms lasted for several weeks. Upon physical examination, it was found that the patient had gout nodules of different sizes in the dorsum of the right hand, middle finger, metacarpophalangeal and interphalangeal joints, confirming gouty arthritis (Figure 1). His left palm, thumb, index finger, middle finger, half of his ring finger, and half of his middle finger were all numb following a neurological examination. During electromyography, the patient's median nerve conduction velocity was decreased, and his sensory function was impaired. The complete blood count, C-reactive protein, and electrolytes were all within normal ranges. A routine laboratory test showed a high level of uric acid (598 mmol/L), and hyperuricemia was diagnosed. He did not take uric acid-lowering drugs. Considering the gout history in this case, we speculated that it was tophaceous gout due to CTS. We arranged an ultrasound (US) examination for the patient. The US confirmed high-density sediment in the patient's carpal canal. To avoid serious complications, operative management was performed when the uric acid level was under control. We took a vertical wrist incision, opened the carpal tunnel, and found some gouty mass deposited under the epineurium of the median nerve (Figure 2). We removed the gouty tophus, rinsed the carpel tunnel with 5% sodium bicarbonate solution, and injected dexamethasone under the epineurium to prevent neuro edema. The postoperative pathology report was consistent with tophaceous gout. In addition to diet structure control and uric acid determination, we strengthened patient health education. The patient's condition improved rapidly after the procedure, and he was discharged home 7 days later. After 1 year of follow-up, his finger range of motion recovered to normal and numbness disappeared.

## Discussion

There is still no consensus on treatment strategies for carpal tunnel syndrome caused by gouty tophi. According to previous research, drugs can be effective in treating CTS caused by gout (13). The early exploration of surgical options has been suggested as a preventative measure when the patient is noncompliant with medical treatment or is displaying severe symptoms. Severe and irreversible complications can be avoided if gout is diagnosed early and managed correctly. Tophaceous gout can affect the flexor tendons and the median nerve within the carpal tunnel. Because the blood of gout patients contains a large amount of urate crystals, these crystals can be deposited throughout the body with blood circulation, such as joints, ligaments, bursa, tendon sheath, subcutaneous tissue, and other parts, and then form gout nodules, resulting in inflammatory reactions such as redness, swelling, fever, and pain. When urate crystals are deposited in the tissue around the carpal tunnel, it can lead to median nerve entrapment and CTS. Gouty lesions should be investigated before surgery in patients with CTS to determine their presence, location, and complexity (2). If the serum uric acid level had been diagnosed earlier and controlled, heavy infiltration of urate crystals into tendons and the nerves might have been prevented and hand function might have been preserved.

The treatment of CTS caused by gout stone includes drug treatment and surgical treatment. Drug treatment can reduce blood uric acid, control the acute attack of gout, and reduce patients’ pain, but the treatment duration is long and the overall treatment effect is not good. What is more, it has no effect on the formed gout stone nor can it prevent it from continuously releasing urate into the blood. In addition, the damage to surrounding tendons and other soft tissues caused by gout stone deposition is irreversible. Medical treatment has been reported to be effective for CTS secondary to gout, but surgical decompression is more reliable and quicker to relieve symptoms, thereby confirming the diagnosis to avoid permanent damage and potential function loss. Therefore, surgical treatment should be the first choice for CTS caused by gout stone to completely remove gout stone, reduce carpal tunnel pressure, and relieve the compression of the median nerve. The surgical treatment and mode selection of CTS have been mature, but due to the particularity of CTS caused by gout stone, the surgical method is different from the conventional operation. The authors tend to combine the comprehensive treatment of internal medicine before the operation and then perform the operation after the patient's blood uric acid decreases to the normal or close to the normal value and is not in the acute attack stage. The choice of surgical incision should be based on the preoperative ultrasonic examination and positioning of the gout stone, and individualized treatment should be taken.

Surgical exploration was required in our case not only to release the transverse carpal ligament and debulk the tophi but also to decompress the median nerve. Moreover, nodular tophus and gouty deposits that had infiltrated the flexor tendons were removed to improve excursion. In some cases, it is difficult to completely remove gout stones during surgery due to their small size and deep deposition. The dissolution effect of gout stones in sodium bicarbonate is better than that of normal saline, so we repeatedly rinsed the incision with 5% sodium carbonate solution to make the gout stones as clean as possible, which is conducive to wound healing and reduce the recurrence rate. Additionally, we recommend releasing the adventitia of the median nerve and injecting dexamethasone to completely decompress the nerve and prevent postoperative edema.

In conclusion, we reported a case of CTS caused by gouty tophi deposited under the epineurium of the median nerve. Despite the surgery being recommended to relieve severe sensory loss, confirm the diagnosis, and decompress the median nerve, gout-related CTS can be treated with medication for hyperuricemia, which is essential in the perioperative period. The control of gout is necessary to avoid disease recurrence. To prevent permanent dysfunction, early diagnosis and control of serum uric acid levels are more important than surgery. If there is evidence of extrinsic compression of the median nerve or symptoms that originate from the carpal tunnel, gouty tophi may be a possible cause.

## Data Availability

The original contributions presented in the study are included in the article/Supplementary Material, further inquiries can be directed to the corresponding author.
